# *Candida auris*: Epidemiology, Diagnosis, Pathogenesis, Antifungal Susceptibility, and Infection Control Measures to Combat the Spread of Infections in Healthcare Facilities

**DOI:** 10.3390/microorganisms9040807

**Published:** 2021-04-11

**Authors:** Suhail Ahmad, Wadha Alfouzan

**Affiliations:** Department of Microbiology, Faculty of Medicine, Kuwait University, P.O. Box 24923, Safat 13110, Kuwait; alfouzan.w@ku.edu.kw

**Keywords:** *Candida auris*, epidemiology, pathogenesis, diagnosis, antifungal susceptibility, environmental contamination, infection control, environmental decontamination

## Abstract

*Candida auris,* a recently recognized, often multidrug-resistant yeast, has become a significant fungal pathogen due to its ability to cause invasive infections and outbreaks in healthcare facilities which have been difficult to control and treat. The extraordinary abilities of *C. auris* to easily contaminate the environment around colonized patients and persist for long periods have recently resulted in major outbreaks in many countries. *C. auris* resists elimination by robust cleaning and other decontamination procedures, likely due to the formation of ‘dry’ biofilms. Susceptible hospitalized patients, particularly those with multiple comorbidities in intensive care settings, acquire *C. auris* rather easily from close contact with *C. auris*-infected patients, their environment, or the equipment used on colonized patients, often with fatal consequences. This review highlights the lessons learned from recent studies on the epidemiology, diagnosis, pathogenesis, susceptibility, and molecular basis of resistance to antifungal drugs and infection control measures to combat the spread of *C. auris* infections in healthcare facilities. Particular emphasis is given to interventions aiming to prevent new infections in healthcare facilities, including the screening of susceptible patients for colonization; the cleaning and decontamination of the environment, equipment, and colonized patients; and successful approaches to identify and treat infected patients, particularly during outbreaks.

## 1. Introduction

*Candida* and other yeast species are part of the microbiome on human skin, mucous membranes, the female genital tract, and the gastrointestinal tract [1,2]. Of nearly 150 *Candida* species described in the literature, only ~10% are known to cause human infections (candidiasis) [3]. The infections range in severity from mild, localized infections (such as vaginitis) to more serious, life-threatening deep-seated invasive infections and candidemia [3,4]. The incidence of candidemia is increasing worldwide, and *Candida* spp. are now recognized as the fourth most common cause of bloodstream/invasive infections, particularly in intensive care unit (ICU) settings in many tertiary care hospitals, where at least 50% episodes of candidemia occur [3,4,5]. *Candida* spp. are also among the four most common causes of late onset septicemia in very-low-birth-weight neonates and infants [6,7]. Major risk factors for invasive *Candida* infections include multiple comorbidities, such as extremes of age, being hospitalized in ICU, total parenteral nutrition, diabetes mellitus, neutropenia, pneumonia or chronic pulmonary diseases, cardiovascular diseases, sepsis, the presence of central venous catheters, urinary tract infection, urinary catheters or acute renal failure, malignancy, prior or concomitant bacterial infection, the use of broad-spectrum antibiotics and antifungal agents, and immunosuppressive therapy [8,9,10,11]. Candidemia has an attributable mortality of 15–35% in adults and 10–15% in neonates [12].

*Candida albicans* is the most common causative agent of candidemia and invasive candidiasis; however, >50% of all infections are now caused by other non-*albicans Candida* species, and their spectrum is rapidly changing [13,14,15,16,17,18,19,20]. Non-*albicans Candida* species have increased in prevalence in many geographical settings, likely due to the increasing use of fluconazole/other antifungal drugs for prophylaxis and therapy. This has resulted in the selection of *Candida* spp. with reduced susceptibility to antifungal drugs, and infections are now associated with higher mortality rates as they often lead to adverse clinical outcomes [19,21,22,23,24,25]. In recent years, we have witnessed an increasing number of reports describing invasive infections by multidrug-resistant *Candida* spp. in various medical centers worldwide [18,19,22,26,27,28]. The emerging multidrug-resistant *Candida* spp. include *Candida glabrata*, *Candida guilliiermondii* complex members, *Candida krusei*, *Candida lusitaniae*, *Candida lipolytica*, *Candida rugosa*, *Candida kefyr*, *Candida haemulonii* complex members, and *Candida auris* [18,19,22,26,27,28,29]. Among these potentially multidrug-resistant *Candida* spp., *C. auris* has attracted a great amount of attention in recent years as it has been linked to major outbreaks of invasive infections in healthcare facilities around the globe [29,30,31,32]. In this article, we describe the current epidemiology of *C. auris* infections and discuss recent approaches to diagnosis, drug resistance, infection prevention, and control measures adopted for *C. auris* to protect susceptible inpatient populations in healthcare facilities.

## 2. Epidemiology of *C. auris* Infections

*Candida auris* is a recently recognized, multidrug-resistant pathogenic yeast that causes invasive infections and outbreaks with high mortality rates in hospitalized patients, particularly among patients with multiple comorbidities and who have been admitted to ICU or other special care facilities [29,30,31,32]. It was first isolated from the external ear canal of a Japanese patient and described as a novel *Candida* species in 2009 [33]. Soon afterwards, 15 other ear isolates collected from 2004 to 2006, which were previously misidentified as *Candida haemulonii*, were described in South Korea [34]. The first six invasive isolates from three patients (including two bloodstream isolates recovered from a 1-year-old girl in 1996) were also described in South Korea in 2011 [35]. Within a decade of its discovery as a novel bloodstream pathogen, >4000 isolates were recovered from blood and other specimens from several countries on all inhabited continents [29,30,31,32,36]. As of 15 February 2021, 47 countries have reported a single case or cluster of cases or outbreaks of *C. auris* infections, according to the Center for Disease Control and Prevention (CDC) of the United States of America (https://www.cdc.gov/fungal/candida-auris/tracking-c-auris.html accessed on 31 March 2021). The epidemiology of invasive *C. auris* infections has seen dramatic changes, as the sporadic invasive infections from the early years have now been replaced by nosocomial outbreaks that are being reported more frequently and appear to involve an increasing number of patients [29,30,31,32,37,38,39]. Studies have shown that once *C. auris* is introduced into a healthcare facility, it spreads rapidly among susceptible patients [40,41]. Thus far, *C. auris* outbreaks have been reported from the United States of America [42,43,44,45], Canada [46], Mexico [47], the United Kingdom [48,49], Spain [50,51], India [40,52], Pakistan [53], Russia [54], Saudi Arabia [55], Oman [56,57], Kuwait [58], Kenya [59], South Africa [60], and Colombia [61]. Studies describing single/multiple invasive infections and outbreaks in different countries or geographical locations in the last several years have been extensively reviewed, only some of which are cited here [29,30,31,32,38]. For a comprehensive listing and chronological order of countries reporting *C. auris* cases between January 2009 and June 2020 and all major outbreaks, readers are directed to two recently published excellent reviews [62,63]. The number of patients affected and the mortality rates in some selected outbreaks reported recently from January 2019 to January 2021 are shown in Table 1. As a result of the increasing incidence of *C. auris* infections, the epidemiology of invasive *Candida* infections has changed dramatically in recent years and *C. auris* has now become a major bloodstream pathogen, even surpassing *C. glabrata* or *C. tropicalis* in some healthcare facilities/geographical settings [41,52,60,64,65,66].

*C. auris* has several unique characteristics, which include its ability to persist, despite the use of common disinfectants, and remain viable for several months, likely due to biofilm formation on plastic surfaces, the hospital environment, and medical devices [68,69,70]. Furthermore, very high rates of resistance to fluconazole and variable susceptibility to other azoles, amphotericin B, and echinocandins make the management of *C. auris* infections extremely difficult [37,56,57,58,71,72,73]. Crude mortality rates varying from 0 to 72% have been reported among *C. auris*-infected patients in different studies [29,30,31,32,37,48,52,56,57,58,74]. *C. auris* frequently colonizes the axilla, groin, nares, respiratory tract, and urinary tract in hospitalized patients [29,30,31,32,58,75,76,77,78]. The environmental screening of patient’s room surfaces and environment including clothing and equipment have yielded *C. auris* isolates with identical fingerprinting patterns, suggesting the shedding of *C. auris* by colonized patients into the environment [48,75,76,77,78,79,80]. *C. auris* has also been shown to persist on reusable skin-surface axillary temperature probes, which coincides with the higher isolation frequency of *C. auris* from the axilla from colonized patients than other body sites [29,30,31,32,44,58,75,76,77,78].

Studies have shown that the rate of *C. auris* colonization in skilled nursing facilities caring for ventilated patients are 10 times higher than its occurrence in nursing facilities without ventilator support [81,82]. The risk factors for the development of invasive *C. auris* infections are similar to those for other pathogenic *Candida* species [29,30,31,32,58,83,84]. Previous studies have shown that *C. auris* colonization results in invasive infections in nearly 10% of colonized individuals, and mechanical ventilation and the placement of invasive devices are two major risk factors for the development of invasive infections due to *C. auris* [48,77,78]. Two recent studies have also shown that other common risk factors for the development of candidemia in *C. auris* colonized patients include total parenteral nutrition, sepsis, longer duration of arterial or central venous catheters, the presence of advanced chronic kidney disease, prior antibiotic use, previous surgery, prolonged ICU stay, and multifocal colonization [65,66]. *C. auris* also has the ability to form ‘dry’ biofilms and aggregative phenotypes which are not easily eradicated [70,77,85,86,87,88]. These characteristics promote the person-to-person transmission of infection through direct/indirect contact in hospital settings rather easily [70,77,85,88].

## 3. Identification of *C. auris* in Culture Isolates and Clinical Specimens

The accurate identification of *C. auris* is crucial for providing optimal patient care, the appropriate treatment of patients with invasive infections, and identifying colonized patients to initiate infection prevention and control measures. *C. auris* isolates are usually misidentified as *Candida haemulonii*, *Candida duobushaemulonii, Candida sake*, *Rhodotorula glutinis*, or other *Candida* species by routinely used phenotypic methods in clinical microbiology laboratories around the world until recently [29,30,31,32,78,89]. At 40 °C, they are able to grow in Sabouraud broth and yeast nitrogen base containing 10% NaCl supplemented with dextrose, dulcitol, or mannitol, while *C. haemulonii*, *C. duobushaemulonii*, *C. albicans*, and *C. parapsilosis* fail to grow under these conditions and *C. glabrata* isolates grow only in Sabouraud broth containing dextrose [44,68]. However, accurate identification by growth at higher temperatures (40–42 °C) or growth in the presence of high (10%) salt concentration are not completely specific for *C. auris* [44,77,90,91,92]. The methods commonly used for the identification of *C. auris* in culture isolates and clinical specimens are summarized in Table 2.

*C. auris* usually forms pink-colored colonies on CHROMagar Candida, and so it is also difficult to distinguish it not only from *C. glabrata* but also from several other *Candida* and yeast species, such as *C. haemulonii* complex members, *Candida kefyr*, *Candida guilliermondii*, *Candida famata*, *Candida conglobata*, and *Candida utilis* which also form pink-colored colonies [29,93]. Furthermore, *C. auris* also undergoes morphological switching between pink, white, and dark purple colony phenotypes when grown on CHROMagar Candida medium [107]. A new chromogenic selective medium, CHROMagar^TM^ Candida Plus has been developed recently; *C. auris* forms distinct cream-colored colonies with a blue halo after 48 h of incubation at 37 °C and is easily differentiated from other *Candida* species, including *C. haemulonii* complex members [94]. CHROMagar Candida medium supplemented with Pal’s medium has also been shown to be useful for the differentiation of *C. auris* from other *C. haemulonii* complex members [108].

*C. auris* isolates were also routinely misidentified, mostly as *C. haemulonii* or *Rhodotorula glutinis*, by automated yeast identification systems such as Vitek2 (Vitek2 YST) until recently [30,34,36,78]. However, Vitek2 YST with upgraded software (version 8.01 which includes *C. auris*) and other automated yeast identification systems now usually identify *C. auris* accurately [41,77,78]. Even then, all clinical isolates identified as *C. haemulonii*, *C. duobushaemulonii*, *C. famata*, and *C. auris* should be confirmed by matrix-assisted laser desorption ionization time-of flight mass spectroscopy (MALDI-TOF MS) or by DNA sequencing (described below) to avoid misidentification. MALDI-TOF MS systems such as Bruker-Daltonics MALDI Biotyper and VITEK MS by bioMeriaux accurately identify *C. auris* only with their updated databases representing all the phylogenetic clades (research use only) or United States Food and Drug Administration (FDA)-approved system databases [39,77,78,95,96,97]. Definitive identification is usually achieved by the PCR amplification of the internal transcribed spacer (ITS) region of rDNA and/or by PCR sequencing of the ITS region or the D1/D2 domains of rDNA [29,30,31,32,41,93,106]. Although whole genome sequencing has been performed to determine phylogenetic relationships among *C. auris* isolates during outbreak investigations, a highly discriminatory, 12-loci-based short tandem repeat typing scheme has also recently been described for the routine fingerprinting of *C. auris* isolates, which yields nearly comparable results [56,58,79,109].

Although automated yeast identification systems such as Vitek2 YST have been improved to correctly identify *C. auris* as stated above, they are slow yielding results in days rather than in hours. More recently, culture-independent methods have been developed for the detection of *C. auris* in few hours to allow the rapid identification of colonized patients. Both in-house [93,99,106] and commercial PCR-based assays [99,100,101,102,104,105] are available. These tests have performed well during clinical evaluations in which culture was used as the gold standard and have yielded >90% clinical sensitivities and specificities. The Taqman qPCR approach has been successfully adapted with the commercial BD Max system for easier, rapid and automated detection of *C. auris* [101,102]. In a recent study, Sattler et al. [104] evaluated the performance of two commercial (*Auris*ID, OLM Diagnostics and Fungiplex *Candida auris* RUO) rt-PCR assays and showed that *Auris*ID assay was more sensitive than the Fungiplex *Candida auris* RUO test. However, *Auris*ID also yielded false positive results, with a high quantity of DNA from other closely related species, while no false positive results were obtained with the other test [104]. Other culture-independent tests have also been developed; however, their performance with clinical samples has not yet been fully evaluated to warrant routine use [93,98,106,110].

## 4. Origin of *C. auris* as a Major Fungal Pathogen and Virulence Attributes

Although *C. auris* was first described in 2009, retrospective analyses of culture collections have identified other *C. auris* isolates obtained previously that were usually misidentified as *C. haemulonii*, including a bloodstream isolate collected in 1996 [34,35]. Phylogenetically, *C. auris* is closely related to *C. haemulonii* complex members [36,111]. Despite highly clonal nature of *C. auris*, whole genome sequence analyses have identified five distinct clades which differ from each other by thousands of single-nucleotide polymorphisms [89,112,113]. These clades are identified by their geographical origin and include South Asian Clade (Clade I), East Asian Clade (Clade II), African Clade (Clade III), South American Clade (Clade IV), and Iranian Clade (Clade V). Interestingly Clade I, III, and IV isolates cause invasive infections and outbreaks frequently, while Clade II isolates exhibit a predilection for the ear which is not normally seen for other isolates [95]. The recently described Clade V isolate was also initially recovered from a case of otomycoses [113,114]. *C. auris* strains exhibit clade-specific resistance to fluconazole, with varying susceptibility to other azoles, echinocandins, and amphotericin B, with many isolates exhibiting a multidrug-resistant phenotype [40,71,73,89,95]. Whole-genome sequence comparisons have also shown that different *C. auris* clades have emerged on different continents nearly simultaneously [89,112,113].

Thermotolerance and salinity tolerance (or osmotolerance) are two unique characteristics of *C. auris* that distinguish it from its close relatives belonging to the *C. haemulonii* complex [29,38,62,63]. Considering the rather recent emergence of this novel yeast pathogen, the simultaneous origin of different clades that differ from each other in thousands of single nucleotides polymorphisms is highly intriguing. It has recently been suggested that *C. auris* initially emerged from a common ancestor, migrated to different geographical locations, and diversified genetically, most likely driven by antifungal prescribing practices [115]. Another study that compared the temperature tolerance of *C. auris* with that of other *Candida* species had suggested that *C. auris* might have previously existed as a plant saprophyte in specialized ecosystems and that climate change, specifically global warming, may have contributed to its ability to grow at higher temperatures and its evolution as a human pathogen (global warming emergence hypothesis) [116]. The authors also suggested a natural wetland or marine (warmer and osmotolerant) environmental niche for *C. auris* [116]. Another study also proposed the aquatic environment as the natural habitat of *C. auris*, as it was used as a prey by two free-living amoebae and proliferated when exposed to protozoal supernatants [117]. Taking a cue from these observations, Arora et al. [118] explored the tropical marine ecosystems around very isolated Andaman Islands, Union Territory of India in the Indian Ocean, and isolated several Clade I *C. auris* from two sites, the virgin habitat of salt marsh area with no human activity and from a sandy beach [118]. The study thus succeeded in isolating *C. auris* for the first time from the tropical coastal environment, outside of the usual hospital environmental settings, suggesting its association with the marine ecosystem. Interestingly, the two isolates from the salt marsh area included a multidrug-susceptible and a multidrug-resistant *C. auris* while all 22 isolates from the beach site were resistant to multiple antifungal drugs [118]. Additionally, all the isolates grew at higher temperatures, however, the multidrug-susceptible isolate from the salt marsh grew slower than other isolates at both 37 °C and 42 °C [118]. Based on the isolation of *C. auris* from tropical marine ecosystems and the observation that one multidrug-susceptible isolate (from salt marsh) grew slower at mammalian temperatures than other environmental (or clinical) strains, Casadevall et al. [119] have suggested that these findings provide an environmental source for clinical isolates and that the common ancestor of *C. auris* has likely adapted to higher temperatures recently. It remains to be seen whether additional environmental *C. auris* will be found from similar ecological sites around the globe that will be more closely related to organisms belonging to other clades.

The virulence factors associated with *C. auris* infections are not completely understood. The adherence of the pathogenic yeasts such as *C. albicans* to the host surface takes place with the help of yeast cell surface adhesion proteins such as agglutinin-like sequence (ALS) proteins (Als1-7, Als9), hyphal wall protein 1 (Hwp1), a glycosylphosphatidylinositol (GPI)-anchored glucan-cross-linked cell wall protein (Eap1) and a GPI-anchored protein 30 (Pga1) [120]. On the other hand, *C. glabrata* relies on epithelial adhesins (Epa) (a subtelomeric gene family) and Epa-like proteins for its attachment to host cells [120]. Genome comparisons have shown that *C. auris* has the capacity to adapt to different environments and possesses many pathogenic mechanisms which are in common with *C. albicans* and other *Candida* species [30,63,89,112,121]. *C. auris* pathogenic attributes that have been identified include pathways required for cell wall modelling and nutrient acquisition, two-component systems, the production of hydrolytic enzymes such as phospholipases and proteinases likely involved in the adherence of the yeast and the invasion of host cells and tissues during infections, other mechanisms of tissue invasion, and immune evasion and multidrug efflux systems [63,89,112,121,122,123]. *C. auris* genome encodes lytic enzymes such as secreted aspartyl proteases (*SAP*s) as well as secreted lipases, phospholipases, and proteases (*YPS*) [121]. *C. auris* genome also encodes orthologs of several *C. albicans* factors that are implicated in adhesion, biofilm formation and virulence [121]. Interestingly, sections of subtelomeric regions that are enriched in putative adhesins are present in outbreak-associated Clade I, Clade II, and Clade IV strains but have been lost by Clade II strains comprising mostly drug-susceptible organisms and associated mainly with ear infections [124]. Other adhesin genes identified in *C. auris* include orthologs of agglutinin, such as sequence (*ALS*)*3* and *ALS4* of *C. albicans*, while the Als3 protein (Als3p) was also detected on *C. auris* cell surface by anti-*C. albicans* Als3p antibodies [63,125]. The *C. albicans* Als3p acts like an adhesin and invasin that mediates attachment to epithelial cells, endothelial cells, and extracellular matrix proteins and induces host cell endocytosis of *C. albicans* hyphae [120,126]. *C. auris* virulence factors and genes conferring resistance to antifungal drugs are presented in Table 3.

*C auris* is also capable of forming biofilms on a variety of surfaces which promote nosocomial transmission. *C. auris* has been cultured from several indwelling medical devices, such as catheters, central/peripheral line tips, and neurological shunts [123,139,140]. *C. auris* forms ‘dry’ biofilms on environmental surfaces and equipment (such as reusable temperature probes) in the hospital and so can remain viable for several months [70,127]. The biofilm-forming ability of *C. auris* has aided its role as a persistent colonizer and difficult to eradicate pathogen from the hospital environment [80,85,86,87,88]. Biofilm formation also protects this organism from antifungal drugs, as was demonstrated by transcriptional analyses and mature biofilms (24 h duration) exhibited resistance to triazoles, polyenes, and echinocandins [128,141]. Kean et al. [128] showed that seven adhesin genes (*IFF4*, *CSA1*, *PGA26*, *PGA52*, *PGA7*, *HYR3* and *ALS5*) are upregulated during biofilm formation. Of these, 4 glycosylphosphatidylinositol (GPI)-anchored cell wall genes (*IFF4*, *CSA1*, *PGA26* and *PGA52*) were upregulated at all (4, 12 and 24 h) time points during in vitro biofilm formation, while two adhesin genes (*HYR3* and *ALS5*) were upregulated only in mature (24 h old) biofilms [128,129]. Furthermore, a number of genes, particularly those encoding efflux pumps such as *MDR* and *CDR* homologs and glucan-modifying enzymes with key role in biofilm extracellular matrix formation were upregulated during biofilm formation, and their inhibition improved the susceptibility of biofilms to fluconazole [128,141,142]. The biofilm-forming capacity of *C. auris* likely has a role in pathogenicity, as many studies have described the clearance of *C. auris* infections in patients after the removal of urinary or central venous catheters [40,50,84,143].

Both morphologic and metabolic plasticity confer an edge for virulence in bacterial and fungal pathogens as this versatility allow the pathogenic organisms to rapidly adapt to different environmental conditions [120,144,145,146]. Different age-dependent phenotypes of *C. auris* have also been described which differ in their susceptibility to antifungal drugs. One study described increased antifungal resistance as a result of transient gene duplication [136]. Compared to younger (0–3 generations) *C. auris* cultures, older (>10 generations) cells exhibited increased tolerance to all four classes of antifungal drugs and older generations of even fluconazole-susceptible cells could survive in very high (up to 256 µg/mL) drug concentration and were unresponsive to fluconazole treatment in *Galleria mellonella* infection model [136]. The decreased susceptibility resulted from both gene duplication and the increased expression of ATP-binding cassette (ABC) transporters (*CDR1*) and lanosterol demethylase (*ERG11*), with older *C. auris* cells showing an 8-fold upregulation of the main azole target gene, *ERG11* [136].

Although metabolic flexibility has been studied in great detail and has been shown to be successfully used by *C. albicans* for virulence [120,146], its role in the pathogenesis of *C. auris* is poorly defined. A recent study has shown that, unlike *C. haemulonii* and *C. albicans,* mannan in *C. auris* is highly enriched in β-1,2-linkages which are important for its interactions with IgG (found in blood and sweat glands) and with the mannose binding lectin (found in blood). The bonding of *C. auris* mannan to IgG was found to be 12- to 20-fold stronger than mannan from *C. albicans*. The findings suggest that the unique mannan of *C. auris* likely has a role in its increased colonization of humans [135]. However, the role of morphologic plasticity has been studied in more detail in *C. auris*.

*C. auris* is a budding yeast; however, some strains fail to release daughter cell after budding, resulting in the formation of aggregates of pseudohyphal-like cells which cannot be disrupted physically or chemically with detergents [123]. *C. auris* isolates producing aggregates of pseudohyphal-like cells were found to be less pathogenic in the *Galleria mellonella* infection model, while non-aggregate-forming *C. auris* strains exhibited pathogenicity comparable to that of *C. albicans* [123]. The ability to aggregate was subsequently shown to be an inducible trait as aggregate formation was stimulated by the prior exposure of *C. auris* to triazoles or echinocandins [130]. A more recent study has shown that the mortality rates between aggregative and non-aggregative *C. auris* strains were nearly same; however, clinical isolates were significantly more pathogenic than reference *C. auris* strains [147]. Aggregative phenotypes of *C. auris* have predominantly been isolated from colonized patients and have higher capacity for biofilm formation than non-aggregative phenotypes, and these findings are consistent with the difficulties encountered in the eradication of *C. auris* from most of the colonized patients [70,88]. On the contrary, in the mouse model of infection, the aggregation of yeast cells has been observed in kidneys of mice that died due to infection, suggesting that aggregate formation may help the yeast to evade immune recognition and thus facilitate its persistence in tissues [139]. Another study has shown that the C5 complement deficiency in A/J mice results in rapid *C. auris* proliferation in target organs, with fatal outcomes, while C57BL/6J mice and mice deficient in neutrophil elastase survive high-dose *C. auris* intravenous challenge, even after cyclophosphamide-induced immunosuppression [131]. These contrasting results are likely due to differences in the virulence of *C. auris* strains tested and/or the infection model.

Although most fungi do not survive at normal physiological temperatures (36.5 °C to 37.5 °C) or during conditions of pyrexia (~40 °C) and are thus unable to colonize humans and cause infections, *C. auris* is capable of growing at higher (>40 °C) temperatures [62,63,68,132]. Similarly, unlike its close relatives (*C. haemulonii* complex members), *C. auris* is also able to tolerate high (>10% NaCl) salt concentrations [63,68,132]. Thermotolerance and osmotolerance are two important characteristics that may help in the persistence and survival of *C. auris* on biotic and abiotic surfaces for long periods of time [48,85,132]. The *Hog1*-related stress-activated protein kinase (SAPK) signaling pathway plays a key role in the *C. auris* response to osmotic stress [129]. The Hog1 SAPK is a highly conserved stress-sensing and signaling protein (*C. auris Hog1* exhibits an 87% sequence identity with *C. albicans* sequence) and a key virulence factor in many human fungal pathogens [124,128]. Day et al. [148] showed that wild-type *C. auris* forms oval yeast cells; however, the deletion of Hog1 resulted in larger elongated cells that clustered together and mutant cells became more sensitive to damage by anionic detergent sodium dodecyl sulphate (SDS). Furthermore, fluorescence microscopy revealed that *hog1* deletion mutants had more exposed chitin indicating that Hog1 plays important roles in cellular morphology, aggregation, and cell wall structure in *C. auris* [148]. Additionally, deletion mutants were sensitive to cationic stress imposed by NaCl or KCl or to high concentrations of sorbitol (osmotic stress). The study also showed that Hog1 is required for the resistance of *C. auris* to the reactive oxygen species (hydrogen peroxide) and to highly acidic environments, but it was dispensable for growth in alkaline and moderately acidic environments and for the resistance to the organic oxidative stress-inducing agents [148]. In the invertebrate model host *Caenorhabditis elegans*, wild-type *C. auris* was more pathogenic than *Hog1*-deleted cells, clearly demonstrating that *Hog1* SAPK is an important pathogenicity determinant in *C. auris* [148]. *C. auris* is known to survive on human skin and environmental surfaces for several weeks to months and is known to tolerate exposure to some commonly used disinfectants. Persistence on surfaces may contribute its transmission within healthcare settings. For instance, the first *C. auris* outbreak in the United Kingdom was linked to the use of reusable axillary temperature probes [48].

In *C. albicans*, phenotypic switching, an adaptation to survive in a harsh environment, is stimulated by several factors, such as exposure to ultraviolet (UV) light, abnormal pH/temperature or nutrient limitation as well as exposure to biological factors present in serum, and involves global changes in gene expression that are controlled by white-opaque regulator (*WOR*)1 [149]. Similar to the yeast-hyphal transition and white-opaque switching observed in *C. albicans*, *C. auris* also undergoes morphological switching between pink, white, and dark purple colony phenotypes when grown on CHROMagar Candida medium likely as a result of distinct cellular oxidative/reductive states [107]. The *C. auris* genome encoded three genes homologous to Wor1 which could potentially control phenotypic switching in *C. auris*. The identification of phenotypic switching in *C. auris* has also led CDC to alert diagnostic facilities to exercise caution when using morphological features for its screening [107]. Phenotypic switching is also observed in *C*. *glabrata*, resulting in four colony phenotypes of white, light brown, dark brown, and very dark brown colonies when it is grown on nutrient agar medium containing copper sulfate or phloxine B [150]. Apparently, *C. auris* colonies undergo this transitioning at a higher rate than the white-opaque switch frequencies observed in *C. albicans* [107]. It remains to be seen whether this phenotypic switching is heritable and also whether it is associated with virulence and/or antifungal drug resistance in *C. auris*.

The formation of true hyphae is another feature of pathogenic *Candida* species which is important for the invasion of host tissue [151]. Although *C. auris* is known to form pseudo-hyphae, it has not been shown to form true hyphae until recently [88,123]. Recent studies have shown that *C. auris* isolates can form true hyphae under certain defined conditions. For instance, the growth of *C. auris* on yeast extract peptone dextrose (YPD) medium supplemented with 10% NaCl induced the formation of elongated/pseudohyphal-like cells at both 37 °C and 42 °C in one recent study [152]. It was further shown that the addition of an Hsp90 inhibitor also led to the formation of pseudohyphal-like cells, similar to Hsp90-mediated temperature-dependent filamentation in *C*. *albicans* [153,154]. Another study based on a systemic infection model has shown that a subset of *C. auris* cells could undergo filamentation after passage through the mouse and three distinct phenotypes (typical yeast cells, filamentation-competent yeast cells, and filamentous-form cells) were detected [133]. Surprisingly, filamentation-competent yeast cells upon subsequent growth on YPD medium at cooler temperatures (<25 °C) showed robust filamentation and were described as “filamentous-form cells” which, under the microscope, looked morphologically similar to true hyphae produced by *C. albicans* [62,133,151]. However, in contrast to the true hyphae and yeast forms of *C. albicans* which are observed at 37 °C and at lower temperatures, respectively [151], lower temperature conditions (<25 °C) promoted while growth at 37 °C repressed filamentous growth in *C. auris* [62,133]. Furthermore, switching between the typical yeast form and the filamentation-competent yeast form, though rare in *C. auris*, was heritable when it did occur while switching between the filamentation-competent yeast cells and filamentous-form cells was nonheritable and dependent on the cooler environment [133]. The yeast and filamentous-form *C. auris* showed differences in global gene expression profiles, the expression of virulence factors, and the increased expression of genes involved in sugar transportation, glycolysis, and energy production, indicating more active metabolism in filamentous cells compared to yeast cells [62,133]. Yue et al. [133] also showed that several genes homologous to *C. albicans* hyphal regulators are upregulated in filamentous cells, suggesting a similarity in the process of filamentation in *C. auris* and *C. albicans*. Their differential expression data showed that G1 cyclin-related protein gene (*HGC1*) and a GPI anchored protein gene (*ALS4*) are upregulated in filamentous form *C. auris* cells. The study further showed that conserved transcriptional regulator-encoding genes (*CPH1* and *FLO8*) that control filamentous growth in *C. albicans* as well as GPI-anchored cell wall related genes *PGA31* and *PGA45* are also upregulated in filamentous-form *C. auris* cells [133]. Interestingly, *WOR1* was downregulated in filamentous form *C. auris* cells [133]. More recently, clinical *C. auris* isolates belonging to all four major clades were shown to form multiple (yeast, filamentous, aggregated and elongated forms) colony and cellular morphologies that differed in antifungal resistance and virulence properties in the *G. mellonella* infection model, suggesting the presence of these features as general characteristics of this organism [134]. Taken together, these studies suggest that filamentous forms of *C. auris* could exist in the cooler hospital environment and perhaps also on the skin of colonized patients, where the temperature could be markedly lower and could be more virulent if they gain access to the inside of the susceptible patients with multiple comorbidities, particularly in ICU settings.

## 5. Susceptibility of *C. auris* to Antifungal Drugs

An important reason for *C. auris* to be known as a “superbug” in recent years is its intrinsic resistance to one, more and sometimes to all available antifungal drugs [30,37,62,73]. Generally, *C. auris* strains exhibit very high rates of resistance to fluconazole and a variable susceptibility to other azoles, amphotericin B, and echinocandins, which makes the antifungal management of *C. auris* infections, particularly invasive infections in patients with multiple comorbidities, extremely difficult [29,30,31,32,39,58,62]. Currently, there are no established susceptibility breakpoints for *C. auris*. However, tentative breakpoints have been suggested by the Centers for Disease Control and prevention (CDC) based on expert opinion and those established for other closely related *Candida* species [62,72,73,89,155,156]. The following tentative breakpoints have been proposed for classifying drug-resistant strains: fluconazole, >32 µg/mL; amphotericin B, 2 µg/mL; caspofungin 2 µg/mL; micafungin 4 µg/mL; and anidulafungin 4 µg/mL.

*C. auris* strains from around the world exhibit a clade-specific resistance to fluconazole but varying susceptibility to other triazoles, amphotericin B, and echinocandins [62,72,73,89,155]. For instance, nearly 90%, 30%, and ~5% of *C. auris* isolates from the USA have been reported to be resistant to fluconazole, amphotericin B, and echinocandins, while the corresponding values for *C. auris* isolates from India have been reported as 90–95%, 7–37%, and <2%, respectively [71,73,79]. Although the resistance rates to fluconazole are usually very high, only a few Clade II isolates are resistant to fluconazole and susceptibility to other triazoles varies widely even among isolates belonging to the same clade [39,58,62,73,95,157]. The current knowledge of genes conferring resistance to antifungal drugs are listed in Table 3. The cytochrome P450-dependent lanosterol demethylase involved in ergosterol biosynthesis and encoded by *ERG11* is the main target conferring resistance to fluconazole [71,73,89]. Three nonsynonymous (F126L, Y132F, and K143R) mutations have been detected in *ERG11* in fluconazole-resistant *C. auris* isolates belonging to different genetic clades with Y132F and K143R being more common [71,73,89]. Although *ERG11* gene mutations are strongly associated with resistance to fluconazole in clinical *C. auris* isolates, their presence alone does not completely explain the entirety of resistance observed clinically, clearly implying the role(s) of other genetic and molecular mechanisms in fluconazole resistance [41,58,71,79,158]. Indeed, recent studies have shown that the molecular basis of resistance to triazoles is much more complex than previously thought. *C. auris* genome encodes several members of the ATP-binding cassette (ABC) transporters (*CDR1*) and major facilitator superfamily (MFS) members (*MDR1*) that coincides with the exceptional multidrug resistance characteristic of this organism and some *C. auris* isolates with K143R mutation in *ERG11* gene were found to contain two copies of the *MDR1* gene encoding for a major facilitator transporter [79,89,136,137,138].

The *CDR1* and *MDR1* homologs are highly expressed in triazole-resistant *C. auris*, and the deletion of *CDR1* causes a >100-fold decrease in the minimum inhibitory concentration (MIC) values for triazoles, suggesting that the overexpression of *CDR1* is a significant contributor to clinical triazole resistance in *C. auris* [137]. Another study showed that both in vitro-generated and clinical fluconazole-resistant *C. auris* isolates contained nonsynonymous mutations in *TAC1B*, encoding zinc-cluster transcription factor and showed increased expression of *CDR1* relative to the parental clinical isolate [138]. Nonsynonymous mutation A640V was detected in *TAC1B* in 57 Clade I isolates containing *ERG11* K143R mutation, nonsynonymous mutation A657V, and frameshift deletion mutation between codons F862 and N866 were detected in 15 and 46 Clade I isolates, respectively, containing *ERG11* Y132F mutation and nonsynonymous mutations K247E, M653V and A651T were detected in 5, 7, and 16 Clade IV isolates, respectively, with no *ERG11* mutations [138]. More importantly, gene replacement studies confirmed the role of the most common (A640V) *TAC1B* mutation as this mutation increased fluconazole MIC 8-fold when introduced into a fluconazole-susceptible strain while the reverse experiment caused 16-fold decrease in fluconazole MIC [138]. Furthermore, a nonsynonymous (G145D) mutation has also been found in *YMC1*, encoding several transmembrane transporter activities essential in mitochondrial transport in some fluconazole-resistant *C. auris* isolates lacking the K143R mutation in *ERG11* [79]. Thus, mutations in *TAC1B* and *YMC1* also contribute to clinical fluconazole resistance in *C. auris*.

Resistance rates to amphotericin B also vary considerably, with one study reporting >60% of *C. auris* isolates as resistant to this polyene drug [39,41,71,89,141]. The molecular basis of resistance to polyenes in *C. auris* is poorly defined [73]. Resistance to polyenes in *C. albicans* and other *Candida* species is mediated by mutations in genes involved in ergosterol biosynthesis, particularly *ERG2* and *ERG6* [28,73,159]. In one study which interrogated *ERG* genes in *C. auris* isolates from the United Kingdom with a reduced susceptibility to amphotericin B, no resistance conferring-mutations were detected [49]. However, Yadav et al. [79] recently showed that all amphotericin B-resistant *C. auris* isolates contain a novel nonsynonymous (G145D) mutation in *ERG2*. Another study has shown the involvement of two-component signal transduction system and mitogen-activated protein kinase (MAPK) signaling pathway in conferring resistance to AMB in *C. auris* [160]. Further studies to elucidate the molecular mechanisms conferring resistance to amphotericin B in *C. auris* are clearly warranted.

Among echinocandins, caspofungin often yields inconsistent results during antifungal susceptibility testing, likely due to paradoxical growth (also known as Eagle effect) of *C. auris* isolates [49,58,72]. The molecular basis of resistance of *C. auris* to echinocandins typically involves nonsynonymous mutations in the hotspot-1 (HS-1) region of *FKS1* encoding 1,3 β-D-glucan synthase [71,72,73]. The most common genetic alteration observed in echinocandin-resistant isolates involves the S639F mutation in HS-1 of the *FKS1* gene [41,71,72,73]. Other nonsynonymous mutations (S639Y and S639P) and the deletion of F635 have also been described in *C. auris* and other *Candida* species isolates with a reduced susceptibility to echinocandins [41,49,58,71,161,162,163].

## 6. *C. auris* Infection Prevention and Control Measures in Healthcare Facilities

Considering the exceptional ability of this organism to cause outbreaks and the very high mortality rates reported among affected patients, specific recommendations and guidelines have been published by the Centers for Disease Control and Prevention (CDC) of USA [164], the European Center for Disease Control (ECDC) [165] and Public Health England [166] for controlling *C. auris* outbreaks in healthcare facilities and are summarized in Table 4.

Major infection control practices include the identification of invasive *C. auris* cases and colonized patients, standard precautions including hand hygiene and personal protection practices, environmental cleaning, and patient decolonization.

### 6.1. Cases of C. auris Fungemia and Other Invasive Infections

As stated above, the risk factors for invasive *C. auris* infections are similar to those for other pathogenic *Candida* species and include immunosuppressed state, multiple comorbidities such as diabetes, hypertension, chronic lung or kidney disease, recent surgery, parenteral nutrition, urinary or central venous catheters, exposure to broad spectrum antimicrobials/antifungals, ventilator support, and stay in ICU settings [29,30,31,32,58,75,76,77]. Although *C. auris*, in addition to fungemia, has also been implicated to cause ventriculitis, pericarditis, complicated pleural effusions and intra-abdominal infections, osteomyelitis, malignant otitis/otomastoiditis, meningitis and vulvovaginitis, its role in respiratory, urinary and skin and soft tissue infections remains uncertain [30,33,34,43,48,50,60,74,89,139,167,168]. Due to its multidrug-resistant nature and extraordinary ability to spread rapidly in healthcare facilities causing outbreaks with associated high mortality rates [30,42,43,44,45,51,52,53,54,55,56,57,58], the detection of even a single case of *C. auris* should trigger an epidemiological investigation and the implementation of infection control measures and contact precautions to prevent further transmission [77,78,141]. This requires the capacity of hospital microbiology laboratory to efficiently and correctly identify *C. auris* and, following the detection of positive cases, the institution of robust infection control measures which include alerting treating infectious disease specialists and notification to institutional authorities for setting up outbreak management teams. It has been observed that delays in the recognition of *C. auris* infection or colonization and delays in the implementation of strict infection control practices typically results in the rapid transmission of *C. auris* among other patients sharing common facilities/equipment [41,48,50,55,56,57,58].

### 6.2. Colonization of Hospitalized Patients with C. auris

Due to the variable case definition and screening practices for *Candida* species, colonization rates and the specific significance of colonization with respect to the development of subsequent invasive infections have been difficult to measure. Although *C. parapsilosis* has been known to cause outbreaks in healthcare facilities [169,170,171], most other *Candida* infections are usually endogenous in origin as opportunistic *Candida* spp. are part of the microbiome in humans [1,2,172]. *C. auris* is highly transmissible among patients, likely due to its tendency to persistently colonize skin and other body sites and is shed into the environment [30,38,41,58,75,79,80,81]. Patients undergoing invasive procedures or the placement of invasive devices are at greater risk of acquiring *C. auris* bloodstream infection with catheters providing an easy access for the fungus to enter bloodstream [38,40]. Colonization has been detected in multiple body sites among outbreak patients and has persisted for >24 months in some patients [41,48,50,58,75]. The anatomic sites usually colonized with *C. auris* include axilla, groin, nose, rectum, respiratory tract, and urinary tract [41,44,48,50,55,56,57,58,79].

A pertinent question that has remained largely unanswered is whether *C. auris* is present in the community or is solely confined to the hospital environment. The screening of new patients for yeasts in general or *C. auris* in particular is not a routine practice in most healthcare facilities, likely due to a lack of perceived importance of yeast colonization. However, few studies have reported on the screening of newly admitted patients for *C. auris* in the hospital settings. The study from the United Kingdom detected *C. auris* in only one among nearly 2200 newly admitted patients, a finding reflecting the low prevalence of *C. auris* infections in newly hospitalized patients [48]. Another study from the USA also detected the organism only in those patients who had previously been exposed to the hospital environment [173]. However, both of the above studies were carried out in countries with a low prevalence of *C. auris* infections. One study from India, a country endemic for *C. auris*, involving a smaller number of patients at a trauma center in New Delhi, did not find *C. auris* in any newly admitted patient [52]. A more recent study has detected *C. auris* at a single site among 3 of 32 chronic respiratory disease patients who were screened at the time of admission to the Chest Diseases Hospital in Delhi, India (and remained colonized at discharge 10–17 days later), and another 9 patients were colonized during their stay in the hospital [79]. Many patients had a history of repeated hospitalization. The study also showed that fomite samples yielded *C. auris* from rooms where colonized patients were admitted nearly 9 days later [79]. *C. auris* has also been frequently isolated from high-touch surfaces, sink drains, and other items from patient’s rooms in centers experiencing *C. auris* outbreaks in the USA, Oman, and Kuwait [56,57,58,69]. Although the previous hospitalization records of the three patients colonized with *C. auris* at the time of admission in the recent Indian study were not reported, it is likely that they were previously exposed to the hospital environment based on the chronic nature of their illness [79]. One study has reported isolation of *C. auris* from the community (swimming pools) in the Netherlands [174]. However, the possibility that the swimming pools were seeded by shedding from individuals who were previously colonized by *C. auris* during their stay in a healthcare or long-term care facility cannot be ruled out. However, contrary to previous efforts, Arora et al. [117] recently succeeded in the isolation of *C. auris* from two environmental sources, the salt marsh area and sandy beaches from Andaman Islands, Union Territory of India, in the Indian Ocean. The findings suggest hot tropical marine ecosystem as one of the natural habitat for *C. auris*. More importantly, the investigators isolated two different strains from a site remote from human activity (the salt marsh area); a multidrug-susceptible and a multidrug-resistant *C. auris* and the multidrug-susceptible isolate grew slower than other isolates at both 37 °C and 42 °C [117]. These findings are consistent with global warming emergence hypothesis put forth recently by Casadevall et al. [116,119], suggesting that *C. auris* likely evolved and adapted to higher temperatures recently.

### 6.3. Transmission-Based Precautions

The shedding of *C. auris* from colonized patients and its transmissibility to other patients in critical care settings within hospitals has been fairly established, and the transmission is facilitated largely due to this organism’s ability to persist in a viable form in the environment around the patient [38,41,48,58,79,80,81]. Viable *C. auris* cells have also been recovered from various environmental sources within the patient’s room/bathroom including beds, bedding materials (mattresses, pillows and bed sheets), bed side trolley, floors, sinks, bathroom door and faucet handles, bathroom walls, medical equipment and disposable/reusable equipment such as oxygen mask, axillary temperature probes and intravenous pole as well as personal mobile phones [48,58,68,69,79]. Cloth lanyards were also found as a source of intermittent transmission of *C. auris* in an ICU in one recent study [80]. *C. auris* has also been shown to survive for weeks on different moist and dry abiotic surfaces such as plastic and steel [68,127,175]. In a recent study, the environmental samples containing *C. auris* colonized patients yielded the organism nearly 7–14 days after colonization was detected [79]. The data are consistent with earlier findings which showed higher rates of *C. auris* colonization in long-term care facilities and co-located hospital and long-term care facilities [81]. In an earlier study, *C. auris* was isolated from the nares of a nurse who provided care to a heavily colonized patient during an outbreak in the United Kingdom [48], and from the hands of two healthcare workers and the groin of another healthcare worker during outbreak investigation in Colombia [176]. Thus, hospitalized and colonized patients, healthcare workers, and contaminated materials could serve as the source for the acquisition of *C. auris* by other hospitalized patients [48,58,68,75,79].

Previous studies have shown that colonization can occur in new patients with a minimum contact time of just 4 h and invasive infections have been acquired by susceptible patients within 48 h of admission to intensive care settings [48,79,177]. Thus, efforts should be made to minimize transmission of *C. auris* to other patients. All *C. auris*-colonized or -infected patients should preferably be placed in a single occupancy room with negative pressure and ensuite bathroom facilities, particularly for those patients with uncontained secretions or diarrhea. Multiple patients colonized or infected with *C. auris* may also be cohorted with other *C. auris* patients, if single rooms are not available [44,48,77,164,165,166]. The rooms housing *C. auris* patients should be clearly flagged to alert healthcare workers and visitors for special precautions and disposable biochemical products and equipment should preferably be used [44,77,164,165,166]. *C. auris* patients should be followed until discharge from the facility and also subsequently for at least one year after they have turned culture-negative during regular screening [77,78]. New patients (with a history of previous stay in a facility known to have *C. auris* cases or colonized patients) should be screened for high-yielding (axilla and groin) and other relevant (urine, throat, wounds, catheter) sites if they are likely to be colonized [77,78,79,175,178,179].

### 6.4. Standard Contact Precautions

Once a *C. auris* case or colonized patient has been detected, it should be immediately reported to the infection control department of the healthcare facility and good standard infection control measures should be immediately instituted [38,77,78,164,165,166]. Patient movement should be allowed only for necessary medical procedures, a minimum number of dedicated healthcare staff should be designated for their care, and the cohorting of staff should be considered for multiple *C. auris* patients [77,78,178,179]. They should be scheduled as the last person for the day on the list for imaging, other procedures or surgery which should be followed by thorough cleaning of the environment. Strict compliance with good hand hygiene before and after touching *C. auris* patients or their surroundings or during medical procedures is essential to prevent transmission of *C. auris* to new patients. Hand washing with soap and water followed by alcohol-based or chlorhexidine-based hand rub has been shown to be effective in eliminating *C. auris* from the hands of healthcare workers [31,180,181,182]. Adequate personal protective equipment (gloves and a long-sleeved gown) must be worn in all contacts with *C. auris* patients or their environment and wearing of a face mask may also be helpful in preventing the colonization of healthcare personnel [48,77,164,165,166].

### 6.5. Environmental and Reusable Equipment Cleaning

The decontamination of the room environment, particularly high-touch areas and medical equipment used in various procedures on *C. auris* patients, is extremely important to prevent further transmission. Although quaternary ammonium compounds (such as hexadecyltrimethylammonium or cetrimide, chlorhexidine, benzalkonium chloride, etc.) are the most commonly used disinfectants in healthcare settings, they have limited activity against *C. auris* [180,182,183]. The twice daily (or three times daily during outbreaks) disinfection of the room environment and high-touch areas in rooms housing *C. auris* patients with active biocides has been shown to be highly effective in controlling further transmission [77,78]. Chlorhexidine shows formulation-dependent efficacy, with one study showing significant killing of *C. auris* cells by chlorhexidine in 70% isopropanol [181].

Sodium hypochlorite at 1000 parts per million (ppm) or higher has been shown to be effective in eradicating *C. auris* during environmental decontamination after patient discharge, though toxicity is a major issue at higher concentrations [42,180,182,183]. However, sodium hypochlorite was effective at higher pH (pH = 11.31) but not at lower pH (pH = 8.68) against dry biofilms containing *C. auris* [127]. Peracetic acid (3500 ppm) and sodium dichloroisocyanurate (1000 ppm) are also effective against dry biofilms containing *C. auris* [127]. Hydrogen peroxide (<1%) or vaporized hydrogen peroxide and povidone-iodine, an antiseptic commonly used for skin disinfection before and after surgery, are also effective [70,183,184,185]. Peracetic acid (3500 ppm, pH 8.82) and sodium hypochlorite (1000 ppm, pH 13.13) have also been shown to be effective in preventing the transfer of *C. auris* after wiping with the disinfectants, while peracetic acid also prevented the regrowth of *C. auris* [127]. Silver nanoparticles (1 to 3 nm in diameter) have recently been shown to be highly effective in a dose-dependent manner against *C. auris* on medical and environmental surfaces, exerting a potent inhibitory activity both on biofilm formation and against preformed biofilms by causing cell wall damage [186]. For small spills, 70% ethyl alcohol is suitable and other products containing ethyl alcohol or phenols may also be effective [70,127,183,184]. Other methods, such as ultraviolet disinfection with proper exposure time, may also be used as an additional safety measure [183,187]. Ready-to-use cleaners and wipes based on hydrogen peroxide, sodium hypochlorite and seven other CDC-approved disinfectants have been shown to be effective against *C. auris* [78]. Terminal cleaning and disinfection of the environment are mandatory when *C. auris* infected or colonized patients are moved from the care area permanently by chemical fogging, vaporized hydrogen peroxide, ozone, chlorine dioxide, ultraviolet light, or titanium dioxide to ensure the disinfection of difficult-to-reach places in patient’s rooms [77,78].

Reusable equipment serves as a source of outbreaks of *C. auris* infection in healthcare facilities [75,77]. If possible, dedicated or single-use devices and equipment should be used for patients infected or colonized with *C. auris*. However, if this is not feasible, equipment and devices should be thoroughly disinfected after every use according to the manufacturer’s instructions and by following the material’s compatibility with the disinfecting agents [75,77,78]. Cleaning procedures should be audited to ensure that re-usable equipment are being disinfected adequately. Equipment and other materials which cannot be disinfected should not be used [75,77,78]. On-site training and auditing to the increase awareness of healthcare workers in infection prevention and control measures with special focus on personal protective equipment and environmental cleaning is also critical to contain *C. auris* [77,78]. Controlling *C. auris* outbreaks in healthcare facilities has proven to be an expensive affair. The total cost of resources to control a *C. auris* outbreak was determined in one study from the United Kingdom. The authors reported that the outbreak control cost exceeded £1 million, and £58,000 was spent every month during the subsequent year [188].

### 6.6. Suppression and Decolonization Procedures for C. auris

There are yet no established protocols for the decolonization of *C. auris*-positive patients. Adherence to central and peripheral catheter care bundles, urinary catheter care bundle and adequate care of tracheostomy site have been advocated to reduce the rate of colonization. Twice daily skin decontamination with 2% chlorhexidine gluconate single-use wash cloths or 4% chlorhexidine solution have been tried in critically ill patients with limited success [48]. Mouth washing with 0.2% chlorhexidine has also been used with patients on ventilator support to reduce oropharyngeal colonization while chlorhexidine impregnated protective disks have been used for central vascular catheter exit sites to reduce line-associated seeding of bloodstream with *C. auris* [48]. However, *C. auris* colonization and further transmission continued to occur in the healthcare facility experiencing the outbreak [48].

Chlorhexidine at standard concentrations with/without alcohol used for skin, and wound cleansing and disinfection and octenidine dihydrochloride are effective only against planktonic *C. auris* populations and not against *C. auris* biofilms [87,181,189]. It has also been suggested that bathing with chlorhexidine may dry the skin which may prolong colonization with *C. auris* [77]. Even if transient decolonization is achieved, recolonization from polyester bedding material on which *C. auris* can survive for several days may lead to persistent colonization in some patients [77,180]. One recent study reported clearance of *C. auris* in 3 of 12 colonized patients (hospitalized for 33–150 days) before their discharge from the hospital; however, the strategies adopted for decolonization were not described [79].

## 7. Treatment of *C. auris* Infections

As stated above, *C. auris* isolates exhibit clade specific resistance to fluconazole, with most Clade I isolates exhibiting high-level of resistance [41,56,57,58,71,89]. Globally, >90% of *C. auris* isolates are resistant to fluconazole, and resistance to amphotericin B can also be >30% in some settings [71,73,89,190]. Consequently, the treatment choices for *C. auris* infections are limited and should be guided by antifungal susceptibility testing results, as resistance rates to amphotericin B and echinocandins also vary in different geographic regions. According to CDC guidelines, consultation with an infectious disease specialist is highly recommended when caring for patients with *C. auris* infection [191]. Echinocandins are recommended as initial therapy for the treatment of invasive *C. auris* infections, which are in line with the general guidelines developed by the Infectious Disease Society of America (IDSA) for the management of candidiasis caused by *Candida* species showing reduced susceptibility to fluconazole [5,191]. The treatment may be changed after 5–7 days from echinocandin to fluconazole if *C. auris* isolate is susceptible to this drug and the patient is stable [5]. However, treatment failures are common and are usually attributed to the development of resistance of *C. auris* to echinocandins, usually due to mutations in *FKS1* gene [41,58,71,72,162]. Liposomal amphotericin B is the usual alternative and voriconazole may also be a suitable choice provided the isolate is susceptible by in vitro susceptibility testing [77]. Only a few studies have reported high success rates during the treatment of *C. auris* infections, mainly facilitated by the low rates of resistance of *C. auris* to antifungal drugs [48,67]. Nearly 4% of *C. auris* isolates are resistant to all presently licensed antifungal drugs, and hence the candidemia cases caused by such strains are potentially untreatable [77,192]. It has also been observed that, despite treatment for invasive infections, patients generally remain colonized with *C. auris* for long periods [41,58,191].

## 8. Conclusions

The emergence of *C. auris* as a major cause of invasive fungal infections in recent years has been dramatic, as is evidenced by the increasing incidence of *C. auris* outbreaks occurring in many countries on all inhabited continents. This fungal pathogen now represents a serious threat to healthcare, as outbreaks have mainly occurred in facilities catering mainly to elderly patients with debilitating comorbidities and are associated with high mortality rates. The outbreaks have been difficult to control, due to its faulty detection by routine diagnostics, rapid transmission, and resistance to removal by environmental disinfection procedures. *C. auris* has now become the leading cause or among the leading causes of invasive fungal infections in many healthcare centers, mostly due to its potential to present or develop resistance to multiple classes of antifungal drugs and due to its ability to persist in healthcare settings. Timely diagnosis by rapid and reliable identification methods and diligence in infection control measures can help to contain the spread of *C. auris* and prevent and control outbreaks.

## Figures and Tables

**Table 1:** Number of patients affected and mortality rates in selected studies reporting outbreaks during January 2019 to January 2021. ^a^ Outcome reported for candidemia patients only ^b^ Clinical details available for only 20 patients; NA, not available.

Country	Outbreak Duration	No. of Patients with *C. auris* Causing			Mortality (%)	Reference
		Candidemia	Colonization	Total		
Kuwait	January 2018–June 2019	17	54	71	36 (50.7%)	Alfouzan et al., 2020 [58]
Mexico	April 2020–October 2020	6	6	12	8 (67%)	Villanueva-Lozano et al., 2021 [47]
Oman	April 2018–April 2019	11	21	32	17 (53.1%)	Al-Maani et al., 2019 [56]
Oman	January 2016–December 2019	23	NA	23	9 (39.1%)	Mohsin et al., 2020 [57]
Russia	January 2017–December 2019	38	NA	38	21 (55.3%)	Barantsevich et al., 2020 [54]
Saudi Arabia	March 2018–June 2019	6	29	35	7 (20%)	Alshamrani et al., 2020 [55]
Spain	October 2017–June 2020	47	287	47	11 (23.4%) ^a^	Mulet Bayona et al., 2020 [51]
USA	May 2018–April 2019	7	5	12	2 (16.7%)	Arensman et al., 2020 [67]
USA	July 2020–August 2020	3	32	35	8 (40) ^b^	Prestel et al., 2020 [45]

**Table 2:** Methods commonly used for the identification of *C. auris* in culture isolates and clinical specimens.

Format	Identification Method	Manufacturer	Turn-Around Time (h)	*C. auris* Misidentified as	Main Reference(s)
Culture-dependent tests	CHROMagar Candida	bioMarieux	24–48	*C. haemulonii/duobushaemulonii*, *C. glabrata*, *C. kefyr*, *C. guilliermondii*, *C. famata*, *C. conglobata*, *C. utilis*	[29,93]
	CHROMagar Candida Plus	bioMarieux	24–48	NA	[94]
	Vitek 2 YST ^a^	bioMarieux	>24	*C. haemulonii*, *C. famata*, *C. lusitaniae*	[30,36,78]
	API 20C AUX		24–48	*C. haemulonii*, *Candida sake*, *Rhodotorula glutinis*^b^	[30,36,78]
	Phenix YIS	BD Diagnostics	~24	*C. haemulonii*, *C. catenulata*	[30,36,78]
	RapID Yeast Plus	Thermo Scientific	>24	*C. haemulonii*, *C. parapsilosis*	[30,36,78]
	MicroScan	Beckman Coulter	~24	*C. haemulonii*, *C. catenulata*, *C. guilliermondii*, *C. parapsilosis*, *C. famata*, *C. lusitaniae*	[30,36,78]
	Vitek MS ^c^	bioMarieux	<12	NA	[39,78,95,96,97]
	MALDI Biotyper ^c^	Bruker Daltonics	<12	NA	[39,78,95,96,97]
	rDNA PCR assay	In-house	4 to 5	NA	[93,98]
	rDNA PCR-sequencing	In-house	8	NA	[29,30,31,32,33,34,35,93]
Culture-independent tests	Taqman qPCR	Roche Diagnostic & Applied BioSystems	4 to 6	NA	[99,100]
	Taqman qPCR	BD Max system	4 to 6	NA	[101,102]
	T2 Magnetic Resonance assay	T2 Biosystems	4 to 6	NA	[103]
	*Auris*ID	OLM Diagnostics	2 to 4	NA	[104]
	Fungiplex *Candida auris* rt-PCR	Bruker	4 to 6	NA	[104,105]
	real-time qPCR	In-house	<8	NA	[106]

^a^ With updated software (version 8.01, bioMérieux, Marcy l’Etoile, France) ^b^ Usual red color is absent. ^c^ With an updated software database that includes *C. auris*; NA, not available.

**Table 3:** *C. auris* virulence factors and genes conferring resistance to antifungal drugs.

*C. auris* Attributes	Encoded Product or Characteristic	Specific Role	Main Reference(s)
Virulence genes or factors	Hemolysin, secreted aspartyl proteinases (*SAP*s), secreted lipases, phospholipase, integrin and adhesins (*ALS3*, *ALS4*)	Adhesion and tissue invasion	[63,89,112,121,122,123]
	Biofilm formation (*IFF4*, *CSA1*, *PGA26*, *PGA52*, *PGA7*, *HYR3*, *ALS5*)	Adherence to surfaces and plastics	[70,86,87,88,127,128,129]
	Aggregating and non-aggregating morphological forms	Adaptation and immune evasion	[86,107,123,130,131]
	Thermotolerance and osmotolerance (*Hog1*)	Survival on biotic/abiotic surfaces	[68,132]
	Phenotypic switching (*Wor1*)	Adaptation and immune evasion?	[107]
	Filamentation-competent yeast cells and filamentous-form cells (*HGC1*, *ALS4*, *CPH1*, *FLO8*, *PGA31*, *PGA45*)	Adaptation and immune evasion	[62,133,134]
	Mannan with β-1,2-linkages	Stronger binding to IgG	[135]
Antifungal resistance genes	Lanosterol demethylase, *ERG11*	Triazole resistance	[41,71,73,89,136]
	F126T, Y132F & K143R mutations		
	Upregulation		
	ATP-binding cassette transporter, *CDR1*	Triazole resistance	[79,136,137]
	Upregulation		
	Major facilitator superfamily member, *MDR1*	Triazole resistance	[79,136,137]
	Upregulation		
	Zinc-cluster transcription factor, *TAC1B*	Triazole resistance	[79,138]
	Gain-of-function mutations		
	Transmembrane transporter, *YMC1*	Triazole resistance	[79]
	Upregulation		
	C-8 sterol isomerase, *ERG2*	Amphotericin B resistance	[79]
	Mutation G145D		
	1,3-β-D-glucan synthase, *FKS1*	Echinocandin resistance	[41,58,71,72,73]
	Hotspot-1 mutations S639F/P, ∆635F		

**Table 4:** Key infection prevention and control steps and recommendations for *C. auris* single case and/or during outbreaks.

Intervention Step	Recommended Actions	Recommendations for Infection Control
Identification of *C. auris* cases	Identify all *Candida* isolates from sterile sites to species level	Notify *C. auris* detection to concerned officials
	Identify species of *Candida* from non-sterile sites if clinically indicated	Alert clinicians and microbiologists
	Identify species of *Candida* from any site from facilities with existing *C. auris* cases	Isolate *C. auris*-positive patients in single rooms
	Identify species of *Candida* from any site from patients with international exposure	Retrospective case-finding
	Confirm *C. auris* identification by updated MALDI-TOF MS or PCR-sequencing of rDNA	
Screening of patients	All patients in close healthcare contact with *C. auris* cases	Alert concerned officials/clinicians/microbiologists
	All new patients previously hospitalized in facilities with *C. auris* cases	Positive patients should be isolated or cohorted
	All new patients with previous admissions in healthcare centers in other countries	Periodic reassessment for the presence of colonization at 1 to 3 months intervals
	Surveillance cultures from axilla, groin, nose, throat, urine, feces, wound drain fluid, insertion sites of venous catheters, respiratory specimens	Two or more assessments, 1 week apart, with negative culture results for deisolation of patients not receiving antifungals
Contact precautions	Place *C. auris*-positive patients in side room possibly with en suite facilities and negative pressure	TBPs enforced till *C. auris*-positive cases remain
	Cohort patients if single room occupancy is not possible, prefer single-use commode	Monitor adherence of HCP to TBPs
	Follow transmission-based precautions (TBPs), including the use of personal protective equipment (PPE) by healthcare personnel (HCP) and prefer single-patient-use items	Appropriate hand decontamination following cleaning of *C. auris*-exposed body fluid/areas
	Special precautions (PPE) to be taken in case of high risk of contact with body/body fluid during the cleaning of *C. auris*-exposed areas	Signage to indicate patients are on TBPs with proper indications for precautions and PPE requirements
	Briefing of both patients and visitors regarding the importance of hand hygiene and TBPs	
Environmental cleaning	Twice or three times (for outbreaks) daily cleaning of room environments with sodium hypochlorite (1000 ppm) or a hospital grade disinfectant effective against *Clostridium difficile* spores	Disinfectants based solely on quaternary ammonium compounds are usually ineffective against *C. auris*
	Prefer single-patient use items (pillows, microfiber cloth for cleaning) and equipment (blood pressure cuffs, temperature probes)	Discard less expensive items that are difficult to decontaminate
	Shared medical equipment should be cleaned and disinfected thoroughly according to the manufacturer’s instructions with terminal cleaning on patient’s discharge	Schedule *C. auris*-positive patients last for imaging, other procedures, and surgeries
	Terminal cleaning of rooms using disinfectants and methods with certified antifungal activity and environmental sampling for *C. auris* culture in an outbreak setting	Monitor environmental and equipment cleaning and adherence to disinfection protocols
	Hydrogen peroxide vapor or ultraviolet disinfection to be used as additional safety measures	Normal cleaning and disinfection should still occur
Hand hygiene	Frequent hand washing by HCP with soap and water, followed by alcohol-based hand rub	Monitor adherence of HCP to hand hygiene practices
Patient decolonization	No established protocols for the decolonization of *C. auris*-positive patients exist	Adherence to central and peripheral catheter care bundles
	Skin decontamination with chlorhexidine body washes, mouth gargles with chlorhexidine in patients on ventilators, chlorhexidine-impregnated pads for catheter exit sites may offer some help	Adherence to urinary catheter care bundle
Education and training of HCP	Education of all HCP including those working with environmental cleaning services about *C. auris* and requirement for appropriate precautions and antibiotic and antifungal stewardship	Monitor adherence to infection control practices and antibiotic and antifungal stewardship

## Data Availability

Data sharing not applicable.
